# Crack-Assisted Charge Injection into Solvent-Free Liquid Organic Semiconductors via Local Electric Field Enhancement

**DOI:** 10.3390/ma13153349

**Published:** 2020-07-28

**Authors:** Kyoung-Hwan Kim, Myung-June Park, Ju-Hyung Kim

**Affiliations:** 1 Department of Energy Systems Research, Ajou University, Suwon 16499, Korea; kkh0217@ajou.ac.kr (K.-H.K.); mjpark@ajou.ac.kr (M.-J.P.); 2 Department of Chemical Engineering, Ajou University, Suwon 16499, Korea

**Keywords:** organic electronics, liquid semiconductors, charge injection, surface engineering, crack engineering

## Abstract

Non-volatile liquid organic semiconducting materials have received much attention as emerging functional materials for organic electronic and optoelectronic devices due to their remarkable advantages. However, charge injection and transport processes are significantly impeded at interfaces between electrodes and liquid organic semiconductors, resulting in overall lower performance compared to conventional solid-state electronic devices. Here we successfully demonstrate efficient charge injection into solvent-free liquid organic semiconductors via cracked metal structures with a large number of edges leading to local electric field enhancement. For this work, thin metal films on deformable polymer substrates were mechanically stretched to generate cracks on the metal surfaces in a controlled manner, and charge injection properties into a typical non-volatile liquid organic semiconducting material, (9-2-ethylhexyl)carbazole (EHCz), were investigated in low bias region (i.e., ohmic current region). It was found that the cracked structures significantly increased the current density at a fixed external bias voltage via the local electric field enhancement, which was strongly supported by field intensity calculation using COMSOL Multiphysics software. We anticipate that these results will significantly contribute to the development and further refinement of various organic electronic and optoelectronic devices based on non-volatile liquid organic semiconducting materials.

## 1. Introduction

Non-volatile liquid organic semiconducting materials have attracted a lot of interest as emerging functional materials for organic electronic and optoelectronic devices in recent years, because these fluidic materials present outstanding advantages such as tunable optoelectronic responses, degradation-free characteristics, solvent-free processability, and ultimate mechanical flexibility and uniformity [1,2,3,4,5,6,7,8]. Various electronic and optoelectronic applications, such as photorefractive devices, organic light-emitting diodes, dye-sensitized solar cells, memory devices, and optically pumped lasers, have been already demonstrated using non-volatile liquid organic semiconductors as active materials [5,6,7,8,9,10,11,12,13,14,15,16]. In particular, it has been reported that fresh liquid organic semiconductors can be continuously injected into the devices through microfluidic channels for preventing performance degradation [15,16]. However, due to the relatively low efficiency in comparison with conventional solid-state devices, considerable research efforts have been devoted to the development of novel molecular structures and device architectures to improve device performance, which is still a challenge [1,11,13,14,15,16,17].

Since charge carriers (i.e., electrons and holes) for operating organic electronic and optoelectronic devices should be essentially injected from electrodes, efficient charge injection into the liquid organic layers is necessarily required to realize high-performance devices based on the non-volatile liquid organic semiconductors [3,17,18]. However, the fluidity of the liquid organic materials causes difficulties in inducing preferable molecular orientations on the electrodes for facilitating charge injection, and intermolecular distances in liquid phases are intrinsically longer than those in solid phases (i.e., less dense molecular packing in liquid phases). Charge injection and transport processes are thus significantly impeded at the interfaces between the electrodes and the liquid organic materials, resulting in overall lower performance [19,20,21,22,23,24,25]. In this context, various methods such as inserting buffer layers and adding ionic dopants have been employed to reduce charge injection barriers and to increase charge carrier concentrations [12,26,27,28,29,30,31,32]. In addition, the charge carrier injection into the organic liquid materials is expected to be significantly improved if the local electric fields inducing the migration of charge carriers near the interfaces are enhanced [18,26,27,33,34]. The electric fields are spontaneously concentrated at the edges of the field plates (i.e., edge effect) [35,36,37,38], where the field intensities are locally increased, and thus the field plate structures (i.e., shapes of electrodes) play a decisive role in the spatial distributions of electric fields. This phenomenon is also applicable to interface engineering for improving the charge injection characteristics of organic electronic devices.

Here we successfully demonstrate efficient charge injection into solvent-free liquid organic semiconductors via cracked metal structures with a large number of edges leading to local electric field enhancement. For this work, silver (Ag) thin films, deposited on deformable fluorinated ethylene propylene (FEP) substrates, were used to generate cracks on the field plates in a controlled manner. The Ag films on the polymer substrates were mechanically gripped and stretched up to fixed ratios, resulting in the formation of the cracks with reproducible patterns [39,40]. Although this simple cracking method can be easily performed to fabricate a large number of edges on metal surfaces without any lithographic processes, heavily cracked metal electrodes normally give a rise to high electrical resistance resulting in performance degradation [41,42,43]. To avoid increases in electrical resistance originating from the structural deformations, the cracked Ag films were transferred and welded onto other intact Ag films to complete the electrode structures, and then the charge injection properties were investigated using a typical non-volatile liquid organic semiconducting material, (9-2-ethylhexyl)carbazole (EHCz) [1,5,6,7,8,10,12,13,14]. The fluidity of EHCz with a glass transition temperature below 0 °C facilitates the penetration of the molecules into the crack structures of the electrodes, clearly showing the effects of the engineered interfacial structures in charge injection. It was found that the cracked structures significantly increased the current density at a fixed external bias voltage via the local electric field enhancement, which was strongly supported by field intensity calculation using COMSOL Multiphysics software. These results suggest great potential for the development and further refinement of various organic electronic and optoelectronic devices based on non-volatile liquid organic semiconducting materials.

## 2. Materials and Methods

### 2.1. Materials

The deformable FEP films with a thickness of 0.125 mm (Teflon® FEP) and EHCz were purchased from Alphaflon (Seoul, Korea) and Sigma Aldrich (Seoul, Korea), respectively, and used as received.

### 2.2. Device Fabrication

**Figure 1:**
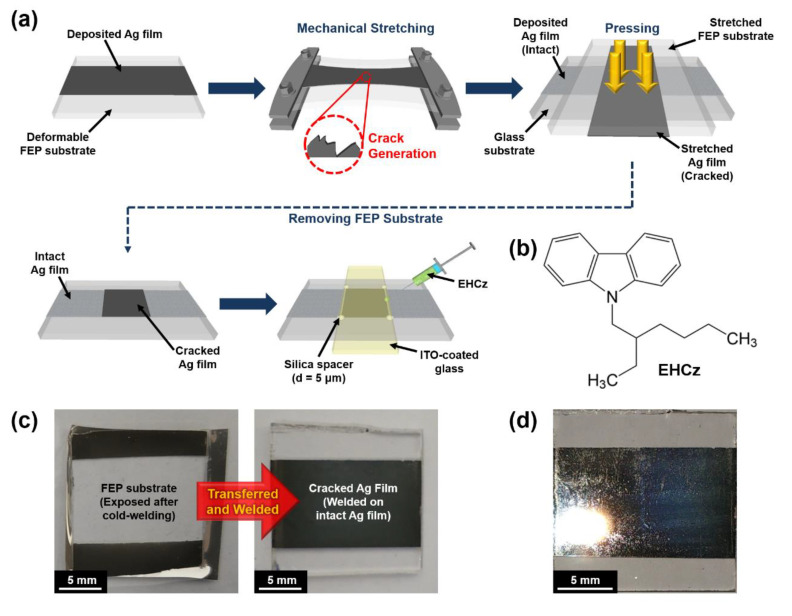
(**a**) Schematic illustration of the preparation of cracked Ag electrode in a controlled manner, and the fabrication of device. (**b**) Chemical structure of (9-2-ethylhexyl)carbazole (EHCz). (**c**) Photographic images of the cracked Ag film after completing the cold-welding process. (**d**) Photographic image of the cracked Ag film inducing diffuse reflection.

The cracked metal electrodes used in this work were prepared as schematically illustrated in Figure 1. The Ag thin films with a thickness of 30 nm were preferentially deposited on the deformable FEP substrates by thermal evaporation in vacuum (<1.0 × 10^−6^ Torr). The samples were individually gripped in a rectangular frame for applying tensile forces, and then uniaxially and biaxially stretched to 120% and 140%, respectively (designated “UA120”, “UA140”, “BA120”, and “BA140”; see also Table 1). It should be noted that the maximum stretching ratio which was reliable without tearing or slipping of the sample in our experimental setup was 140%. To prevent an increase in electrical resistance, each cracked Ag film was transferred and welded onto another intact Ag film by means of cold-welding [44,45,46,47]. For the cold-welding process, the stretched Ag/FEP sample was brought into contact with the intact Ag film (with a 30-nm thickness) deposited on a glass substrate, and subsequently pressed with a pressure of 0.2 MPa for 90 s at room temperature. The FEP substrate was then easily peeled off from the sample without residue owing to a lower surface energy of FEP (see Figure 1c). All the transferred samples exhibited no significant change in high electrical conductivity, compared to a reference electrode (i.e., the intact Ag film with a 60-nm thickness deposited on the glass substrate). It was also confirmed that the cracked Ag films were neatly transferred onto the intact Ag films, using scanning electron microscopy (SEM).

**Figure 2:**
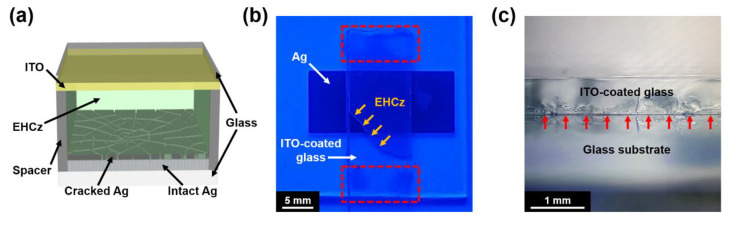
(**a**) Configuration of device to investigate the charge injection properties via local electric field enhancement. (**b**) Photographic image of the device illustrated in (**a**), under 365 nm UV light. The optically excited EHCz material under UV irradiation clearly revealed its liquid boundary during the capillary action (indicated by yellow arrows). Red dashed boxes indicate the locations of silica microsphere spacers. (**c**) Optical microscopy image of a cross section of the device in (**b**). The gap between the two electrodes is indicated by red arrows.

Each prepared Ag electrode was covered with another glass substrate coated with indium-tin oxide (ITO) (20 Ω sq^−1^), and silica microsphere spacers (of 5-μm diameter) were used for a fixed gap distance between Ag and ITO. The gap between the two electrodes was then filled with EHCz by capillary action to complete the device structure (see Figure 2). The active area of each device was 1 × 1 cm^2^. It is worth noting that EHCz is highly viscous, which substantially hinders the infiltration into smaller gaps (in the submicron range). Even if EHCz well stays in the gap without leakage due to its high viscosity, the two substrates (i.e., lower and cover glasses) should be securely fixed to prevent slipping by the liquid.

### 2.3. Measurements

The current density-voltage (J-V) characteristics of the devices were measured in response to a voltage sweep from 0.0 to +1.0 V, using a Keithley 2636 source meter. The capacitance of EHCz was also measured with Agilent 4284A Precision LCR Meter (Agilent, Santa Clara, CA, USA), to evaluate the dielectric constant.

### 2.4. Field Intensity Calculation

The field intensity within the device was calculated using COMSOL Multiphysics software (Burlington, MA, USA). Model structure of the device was prepared, based on the experimental observation as will be discussed in the Section 3. Static analysis was also performed to elucidate the spatial distribution of local electric fields within the device at a fixed bias voltage of +1.0 V.

## 3. Results

**Figure 3:**
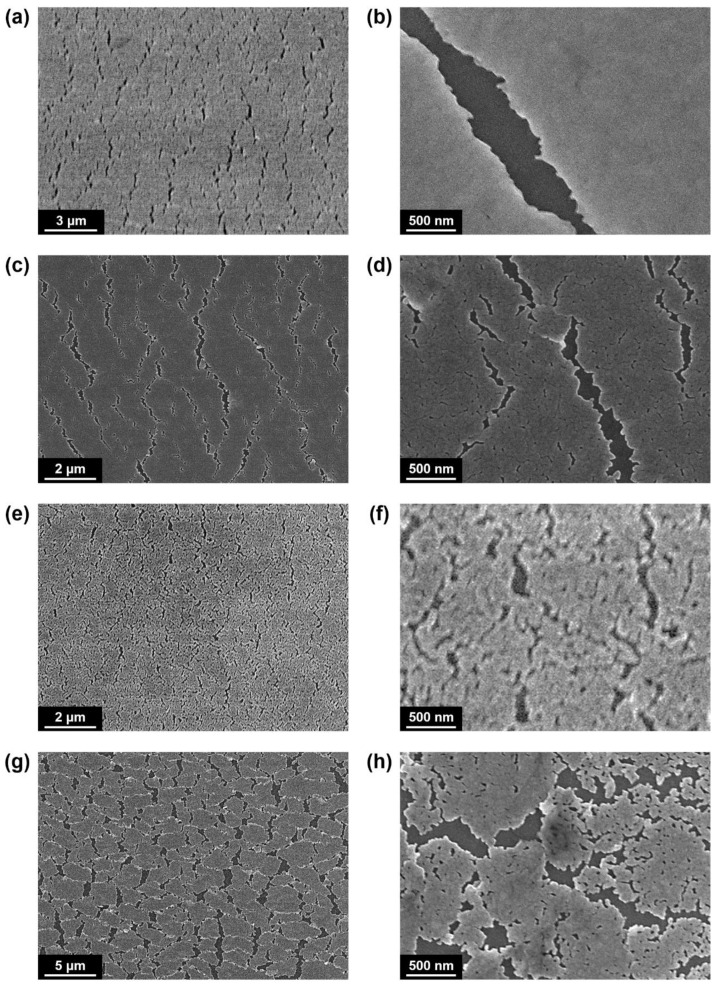
SEM images of the cracked Ag electrodes after completing the cold-welding processes. (**a**,**b**) SEM images of the UA120 sample. No further deformation of Ag was observed between crack lines as in (**b**). (**c**,**d**) SEM images of the UA140 samples. Minor cracks were further observed between major crack lines as in (**d**). (**e**,**f**) SEM images of the BA120 sample. Orientations of cracks were significantly diversified in comparison with the uniaxially stretched samples as in (**f**). (**g**,**h**) SEM images of the BA140 sample. The Ag film was divided into island forms as in (**g**), and minor cracks with a high density were clearly observed within the Ag islands as in (**h**).

**Figure 4:**
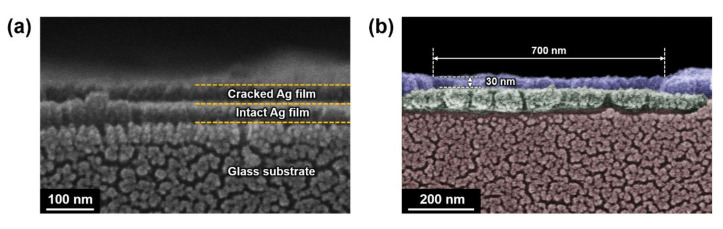
(**a**) SEM image of a selected cross section of the BA140 sample after completing the cold-welding process. (**b**) Colored SEM image of a selected cross section of the same sample as in (**a**). Red, green, and blue regions represent the glass substrate, the intact Ag film, and the cracked Ag film, respectively. The cross section of the crack (with 700 nm width and 30 nm depth) is clearly revealed in the blue region.

The prepared Ag electrodes via mechanical stretching exhibited a matt dark gray color, because the cracked metal structures cause diffuse reflections rather than specular reflections (see Figure 1d). To confirm the overall shapes of the cracks formed on the Ag electrodes, the samples after completing the cold-welding processes were investigated using SEM as shown in Figure 3 and Figure 4. For the UA120 and UA140 samples (i.e., uniaxially stretched to 120% and 140%, respectively), the Ag films tended to crack in the form of a one-dimensional line. With an increase in the uniaxial stretching ratio, the overall crack size increased, and the edges of the cracks were more clearly observed. Particularly, in the UA140 sample, minor cracks were further observed between the major cracks, leading to a higher crack density compared to UA120 (see Figure 3d). For the BA120 sample, which was biaxially stretched to 120%, the orientations of the cracks were significantly diversified in comparison with the uniaxially stretched samples. The overall shape and density of the cracks were comparable to the minor cracks of UA140; however, large cracks similar to the major cracks of UA140 with the well-defined edges were not observed in BA120. As the biaxial stretching ratio increased up to 140% (i.e., BA140), the Ag film was eventually divided into island forms by major cracks, and an average area of the islands was found to be less than 3 μm^2^. In comparison with the other samples, minor cracks with a higher density were clearly observed within the Ag islands as shown in Figure 3h.

Figure 4 also shows cross-sectional SEM images of the BA140 sample. For these SEM measurements, platinum (Pt) was coated onto the sample with a thickness of ~5 nm to clearly observe the non-conductive glass substrate. It was confirmed that the cracked Ag film was neatly transferred and welded on the intact Ag film through the cold-welding process. It is worth noting here that metal oxide layers could not be easily transferred and welded onto other substrates in our experimental setup, and thus binding materials would be required to enhance adhesion.

**Figure 5:**
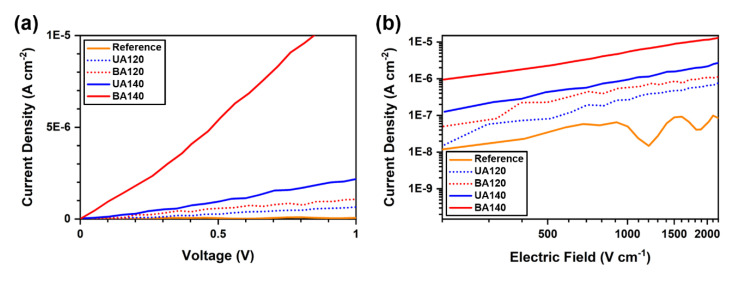
(**a**) Current density-voltage (J-V) characteristics of the devices using the reference, UA120, UA140, BA120, and BA140 electrodes, in a linear scale. The characteristics were measured in low bias region (i.e., ohmic current region). All the characteristics showed a linear relationship, and that the slope ratio of the device with the BA140 electrode to the reference devices was ~170. (**b**) Electric field dependence of the current density. All the slopes were measured to be ~1, indicating ohmic current in each device.

The J-V characteristics for the devices were measured in response to a voltage sweep from 0.0 to +1.0 V, as shown in Figure 5. It should be noted that the charge carriers are normally accumulated in the organic layers in high bias voltage region, due to the relatively low charge carrier mobility. Such charge carrier accumulations in the organic layers give a rise to changes not only in the electric field distributions, but also in the J-V characteristics (i.e., from ohmic currents to space-charge-limited currents) [17,26,28,48,49]. Thus, changes in the charge injection properties according to the local electric field enhancement can be more clearly examined in low bias region (i.e., ohmic current region). As indicated in Figure 5, the current density was gradually increased as the density of the cracks on the Ag electrode increased. In particular, it was found that the slope ratio of the device with the BA140 electrode to the reference devices was ~170 in the J-V characteristics. At a fixed bias voltage of +1.0 V, the current densities were measured to be 6.68 × 10^−8^, 6.60 × 10^−7^, 1.08 × 10^−6^, 2.20 × 10^−6^, and 1.16 × 10^−5^ A cm^−2^ for reference, UA120, BA120, UA140, and BA140, respectively. It is worth noting that EHCz is an intrinsically p-type material, of which conduction is entirely governed by holes [1], and the devices presented in this work can be described as hole-only devices.

All the devices showed linear J-V characteristics within the bias voltage range (i.e., 0.0 to +1.0 V), indicating the ohmic behaviors of the devices in low bias region. If there is no change in charge carrier mobility, the ohmic currents of organic electronic devices at constant temperature are normally enhanced as the initial concentrations of charge carriers within the semiconducting layers increase using ionic dopants [31,32]. However, in this work, the increases in the ohmic currents were solely induced by the injected charges from the electrodes without using any extra dopants. These results strongly suggest that efficient charge injection via local electric field enhancement can exert similar effects to the introduction of ionic dopants on J-V characteristics in terms of charge carrier concentrations. It is notable that the maximum ohmic current density of 1.16 × 10^−5^ A cm^−2^ in this work is relatively lower than those of other solid-state devices based on carbazole derivatives or similar organic semiconducting materials. In ITO/undoped organic semiconductor/metal structures, previous works reported ohmic current densities of ~10^−4^ A cm^−2^ for 4,4′,4′′-tris(N-3-methylphenyl-N- phenyl-amino)-triphenylamine [32], poly(2,6-diphenyl-4-((9-ethyl)-9*H*-carbazole)-pyridinyl-*alt*-2,7-(9,9-didodecyl)-9*H*-fluorenyl) [50], and N,N′-bis(3-methylphenyl)-N,N′-diphenylbenzidine [51], and even higher ohmic current densities were also found with a few of carbazole derivatives [52]. In addition, a few photoluminescent devices were successfully demonstrated using EHCz as a host material in the previous studies [5,6,8]; however, at the current stage, we could not observe electroluminescence from dye-doped EHCz materials due to the imbalance of electrons and holes.

**Figure 6:**
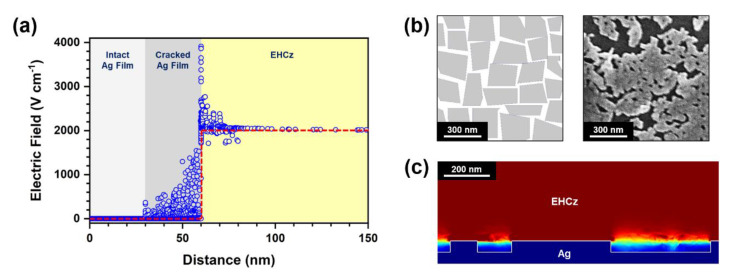
(**a**) Local electric field intensities according to the distance from the Ag bottom, based on the field intensity calculation at a fixed bias voltage of +1.0 V. Blue circles indicate the local electric field intensities in the device with a highly cracked Ag structure (30-nm depth), and a red dashed line indicates the electric field in the reference device. (**b**) Model structure configured for the field intensity calculation in (**a**), and SEM image of BA140 used to prepare the model structure. (**c**) Local electric field intensities visualized for a selected cross section. The boundary between Ag and EHCz is indicated by a white line. The intensity level, presented by colors, increases from blue to red (i.e., blue-green-yellow-red).

The field intensity calculation was also performed to investigate the local electric field enhancement induced by a highly cracked metal structure as shown in Figure 6. The cracked Ag structure with a depth of 30 nm was considered for the calculation, on the basis of the observed SEM image (see Figure 6b). In the simulation model, the gap distance between the lower Ag and upper ITO electrodes was fixed at 5 μm, and the gap was filled with a dielectric material corresponding to EHCz. For the dielectric material, dielectric constant of 3.02, electrical conductivity of 2.1 × 10^−9^ S cm^−1^, and density of 1.004 g mL^−1^ were used as material parameters to simulate EHCz. It should be noted that the dielectric constant of EHCz was experimentally measured for this work. For the measurement, EHCz was injected into the gap between two Ag electrodes with a gap distance of 5 μm, of which the capacitance was monitored at a frequency of 1 kHz. The measured capacitance was converted into the dielectric constant in consideration of active area and thickness.

Static analysis was performed to clarify the spatial distribution of local electric fields within the device at a fixed bias voltage of +1.0 V. According to the distance from the Ag bottom, the local electric field intensities were calculated for the selected cross sections, as shown in Figure 6a. The local electric field intensities were significantly enhanced within the cracks of the Ag electrode, of which apexes were found at the upper edges. In particular, the intensity of the local electric field increased up to ~4000 V cm^−1^ at the upper edges. To clearly show the local electric field enhancement, the field intensities for the selected cross section were further visualized as shown in Figure 6c. These calculation results were in good agreement with the experimental results, where charge injection properties were dramatically improved by the high-density cracks contributing to local electric field enhancement.

## 4. Summary

We demonstrate efficient charge injection into solvent-free liquid organic semiconductors via cracked metal structures with a large number of edges leading to local electric field enhancement. For this work, the Ag thin films on the deformable FEP substrates were mechanically stretched to generate the cracks on the surfaces in a controlled manner, and subsequently transferred and welded onto other intact Ag films for avoiding increases in electrical resistance. Using the prepared Ag electrodes with varied crack densities, the charge injection properties into EHCz were investigated in low bias region (i.e., ohmic current region). It was found that the device with the highly cracked electrode dramatically increased the current density within the ohmic current region, indicating that efficient charge injection via local electric field enhancement can exert similar effects to the introduction of ionic dopants on J-V characteristics in terms of charge carrier concentrations. Although the hole-only devices were demonstrated in this work, these results still offer a wide range of possibilities for various device applications. Careful consideration of work functions of electrodes and cascade energy levels would be required for optimizing the device design or selection of device components. In addition, the field intensity within the device was calculated using COMSOL Multiphysics software, based on the experimental observation, and static analysis was also performed to reveal the spatial distribution of local electric fields. The calculation results were in good agreement with our experimental results, where charge injection properties were dramatically improved by the high-density cracks contributing to local electric field enhancement. We anticipated that these results will significantly contribute to the development and further refinement of various organic electronic and optoelectronic devices based on non-volatile liquid organic semiconducting materials.

## Figures and Tables

**Table 1:** Stretching ratios of Ag films.

Sample Name	Stretching Ratio (X-Axis)	Stretching Ratio (Y-Axis)
Reference	0%	0%
UA120	20%	0%
UA140	40%	0%
BA120	20%	20%
BA140	40%	40%

X- and Y-axes are in-plane, which are perpendicular to each other.
